# Quality of Life in Patients with CKD With Catastrophic Health Care Expenditures: A National Study From Thailand

**DOI:** 10.1016/j.xkme.2025.100987

**Published:** 2025-02-27

**Authors:** Pornpen Sangthawan, Sarayut L. Geater, Pinkaew Klyprayong, Pimwara Tanvejsilp, Sirirat Anutrakulchai, Pongsathorn Gojaseni, Charan Kuhiran, Pichet Lorvinitnun, Kajohnsak Noppakun, Watanyu Parapiboon, Adisorn Pathumarak, Supinda Sirilak, Pleumjit Tankee, Puntapong Taruangsri, Piyamitr Sritara, Nathorn Chaiyakunapruk, Chagriya Kitiyakara

**Affiliations:** 1 Department of Medicine, Prince of Songkla University, Hat Yai, Songkhla Thailand; 2 Department of Medicine, Ramathibodi Hospital, Mahidol University, Bangkok, Thailand; 3 Department of Social and Administrative Pharmacy, Prince of Songkla University, Hat Yai, Songkhla, Thailand; 4 Department of Medicine, Khon Kaen University, Khon Kaen, Thailand; 5 Department of Medicine. Bhumibol Adulyadej Hospital, Directorate of Medical Services, Royal Thai Air Force, Bangkok, Thailand; 6 Department of Medicine, Somdej Pranangchao Sirikit Hospital, Sattahip, Chonburi, Thailand; 7 Department of Medicine, Sunpasitthiprasong Hospital, Ubonratchathani, Ubonratchathani, Thailand; 8 Department of Internal Medicine, Faculty of Medicine, and Pharmacoepidemiology and Statistics Research Center, Faculty of Pharmacy, Chiang Mai University, Chiang Mai, Thailand; 9 Department of Medicine, Maharat Nakhonratchasima Hospital, Nakhonratchasima, Thailand; 10 Department of Internal Medicine, Naresuan University, Phitsanulok, Thailand; 11 Department of Medicine, Vachiraphuket Hospital, Phuket City, Phuket, Thailand; 12 Department of Internal Medicine, Nakornping Hospital, Chiangmai, Chiangmai, Thailand; 13 Department of Pharmacotherapy, College of Pharmacy, University of Utah, Salt Lake City, Utah; 14 IDEAS Center, Veterans Affairs Salt Lake City Healthcare System, Salt Lake City, UT

**Keywords:** Asia, catastrophic health care expenditures, chronic kidney disease, dialysis, economic, financial, health inequity, quality of life, renal, socioeconomic, Thailand, universal health care

## Abstract

**Rationale & Objective:**

Despite universal health coverage, patients with chronic kidney disease (CKD) in middle-income nations still face financial hardship. Catastrophic health care expenditures (CHEs) serve as a valuable index of patient-derived financial hardship, but few studies have explored the connection of CHE with clinical correlates, especially in patients with CKD. This study aimed to assess the association between CHE and health-related quality of life (HRQoL) in a spectrum of patients with CKD in Thailand.

**Study Design:**

A multicenter, nationwide cross-sectional study.

**Setting & Population:**

Patients with CKD (stages 3-5 and dialysis) from 11 centers across Thailand.

**Exposures:**

Catastrophic health expenditures.

**Outcomes:**

Health-related quality of life.

**Analytical Approach:**

Data on clinical, socioeconomic status, and out-of-pocket expenses were acquired via interviews. The CHE was defined as health care expenditures of at least 40% of the household’s capacity to pay. The HRQoL was assessed using the EuroQol-5 Dimensions (EQ5DL) questionnaire. Fractional and multivariable logistic regression models were used to determine the CHE’s effect on EQ5DL composite utility scores and each HRQoL dimension.

**Results:**

Of 1,224 patients with CKD, 20% experienced CHE. EuroQol-5 Dimensions utility scores were notably lower in those with CHE (CHE, 0.76 vs No CHE, 0.82, *P* < 0.001) after adjustments for confounding factors. Differences between CHE and non-CHE appeared in mobility, self-care, and usual activity, with multivariable analysis showing more severe mobility and activity issues in CHE. (adjusted OR [95% CI] in CHE vs non-CHE: mobility: 1.89 [1.23-2.91], *P* = 0.004; usual activity: 1.82 [1.10-3.02], *P* = 0.020].

**Limitations:**

Cross-sectional design prevents causal inferences.

**Conclusions:**

Despite health coverage, patients with CKD with financial strain experience reduced quality of life, with pronounced effects on mobility and daily activity. Integrating the assessment of patient-derived financial burden is an essential step into CKD care plans in middle-income countries.

## Plain Language Summary

Patients with chronic kidney disease (CKD) often face substantial out-of-pocket payments for their care. This study aimed to understand how catastrophic health care expenditures (CHE) affect CKD patients' quality of life. CHE occurs when medical costs overwhelm a household's finances. We conducted a nationwide study in Thailand to examine how CHE affects patients with CKD’s well-being, focusing on mobility, daily activities, and emotional health. By analyzing data from patients across different stages of CKD, we found that patients experiencing CHE reported significantly lower quality of life than those without CHE. Our findings identify the need to address financial hardship in CKD care, emphasizing integrated support strategies such as financial planning and assistance to reduce the burden of CHE on daily life.

Chronic kidney disease (CKD) is a major noncommunicable disease with global health and economic implications.^1^, ^2^, ^3^ Patients with CKD often face substantial out-of-pocket expenses, with individuals in low-income and middle-income countries (LMICs) bearing a heavier financial burden than those in high-income countries, where public funding for care is more available.^4^^,^^5^ As CKD progresses from nondialysis to kidney failure, the financial strain on patients and families worsens.^3^^,^^4^ The Global Kidney Health Atlas survey found that 61% of patients treated with hemodialysis (HD) in high-income countries make out-of-pocket payments, when compared with 77%-83% in LMICs.^5^ Financial risk protection is crucial to prevent patients with CKD from falling into poverty because of health care costs, a key objective of universal health coverage and the United Nations sustainable development goal.^6^, ^7^, ^8^

Catastrophic health care expenditures (CHEs) serve as an indicator of inadequate financial protection in health care.^9^, ^10^, ^11^ The CHE is defined as medical out-of-pocket spending exceeding a specific capacity-to-pay threshold (typically 10%-40%), leading households to forego nonmedical necessities, increasing risks of declining health status, poverty, and socioeconomic disparities. Unlike conventional socioeconomic indices, CHEs directly assess healthcare expenses’ impact on households, providing insights into financial hardship and its influence on access to care. The CHE is also a reliable indicator of health inequity, capturing variations between households with similar incomes because of disparities in health needs and the quality of health care services.^11^^,^^12^

Financial burden depends on the level of health care support, underlying severity of chronic illness, and income.^12^ In LMICs without public funding, the prevalence of catastrophic health expenditures may reach 90% among patients treated with HD, and the extreme financial toxicity from out-of-pocket payments prevents starting or continuing vital dialysis.^13^^,^^14^ In middle-income countries with universal coverage, residual out-of-pocket costs for uncovered aspects of CKD care payments may still impose significant financial strain. Thailand, an upper-middle-income country with 70 million people, exemplifies this issue. Before 2000, economic crises led to a higher financial burden associated with decreased health care utilization and increased mental stress among Thais.^15^ The Thai government launched the Universal Coverage Scheme (UCS) in 2002 for previously uninsured individuals who were not covered by the other 2 state schemes: the Social Security Scheme (SSS) and the Civil Servant Medical Benefit Scheme (CSMBS).^15^ The UCS extended coverage for dialysis in 2008 under the peritoneal dialysis (PD) First Policy, mandating PD as the first treatment unless contraindicated.^14^ Both HD and PD are reimbursed under the SSS or CSMBS. Although all healthcare schemes cover essential medications and kidney replacement, patients must still bear varying out-of-pocket expenses for different treatments.^16^, ^17^, ^18^ Thailand now has 98.5% universal coverage for kidney replacement therapy,^4^^,^^5^^,^^14^^,^^19^ but uncovered out-of-pocket payments continue to lead to significant CHEs, affecting as many as 50% of patients treated with HD.^20^

Patients with CKD experience reduced health-related quality of life (HRQoL), a multidimensional concept identified by the WHO and patients with CKD as a key outcome measure.^21^^,^^22^ Although relationships between HRQoL and healthcare system costs have been demonstrated,^23^ limited data exist on the relationship between financial hardship from a patient’s perspective and HRQoL in CKD. By evaluating out-of-pocket costs and the capacity to pay, CHE is considered a valuable index for assessing the impact of financial burden on health and stress.^9^ However, few studies have explored the connection between CHEs and clinical outcomes, with most focusing on cancer or general populations.^24^^,^^25^ A study from India examined CHEs and HRQoL in patients with CKD but had limitations, such as a small sample size, focusing only on patients treated with HD, and evaluating only the composite utility score without investigating individual dimensions of HRQoL.^26^

Whether catastrophic health expenditures are associated with decreased quality of life across the CKD spectrum and which HRQoL dimensions are most affected has not been fully explored. This multicenter, nationwide cross-sectional study investigates the relationship between CHEs and HRQoL among Thailand's nondialysis, HD, and patients treated with PD. Patients with nondialysis CKD face consequential medical expenses, potential income loss, and quality-of-life limitations. In addition to these challenges, patients treated with dialysis also have greater treatment-related payments and lifestyle restrictions, exacerbating financial and emotional burdens. By including all groups, we aim to provide a broad view of financial hardship across CKD stages, offering a novel perspective into the connection between CHEs and HRQoL in the context of a middle-income country with universal health care.

## Methods

### Study Design

This cross-sectional multicenter nationwide study is reported by following the STROBE Statement.^27^ This study followed the ethical standards outlined in the 1964 Declaration of Helsinki and its later amendments. All participants provided informed written consent, and the study protocol was approved by the Institutional Review Board of the Central Research Ethics Committee (ID: COA-CREC 005/57) and the Faculty of Medicine Ramathibodi Hospital (ID: MURA2023/616).

### Study Population

We conducted this study in 11 tertiary or regional hospitals covering all 5 regions in Thailand from June 2019 to January 2021 as part of the CORE-CKD study (TCTR20211209001) (www.thaiclinicaltrials.org).^20^ The study population consisted of 4 groups of patients with CKD aged 18 years or older: CKD15-60 (stages 3-4), CKD<15 (stage 5, but not treated with dialysis), PD, and HD. The exclusion criteria included current participation in an intervention trial, a previous kidney transplant, or reduced life expectancy of fewer than 3 months because of conditions such as advanced cancer or cirrhosis.

We randomly selected specific periods for patient enrollment from each hospital. During those periods, consecutive sampling was used to enroll patients (n = 100-200 from each hospital), ensuring that all eligible individuals presenting at the hospital during that time were included. We collected demographic, clinical, and socioeconomic data through structured face-to-face interviews with patients and caregivers conducted by a trained study team. The interviews included detailed questionnaires on various expenditures, and a medical chart review supplemented the data collection process. The estimated glomerular filtration rate was calculated using the CKD-Epidemiology Collaboration equation.^28^ Patients were categorized by health insurance schemes with different benefit packages: UCS, SSS, and CSMBS.^17^^,^^28^^,^^29^ We excluded patients with incomplete expenditure data or those who were entirely self-paid.

#### Socioeconomic Data

Socioeconomic data included patient income, food expenses, and total household consumption spending within one month preceding the interview. Out-of-pocket expenditures within 6 months before the interview were collected and categorized into medical or nonmedical. Medical out-of-pocket expenditures included excess copayments for medications or procedures, which the health schemes did not cover. Nonmedical out-of-pocket expenditures included food, transportation, accommodation during clinic visits and hospital admissions, home renovations, or patient care expenses. The annual expenditures were calculated in Thai baht, adjusted with the cumulative inflation rate from the data collection to 2022, and then converted to US dollars using the exchange rate in November 2022.

#### Catastrophic Health Expenditures

Patients were classified as having CHE if their health care expenditures were at least 40% of the household’s capacity to pay, according to a definition used by WHO.^11^ Capacity to pay was defined as the effective income (based on total household expenditures) after subtracting basic subsistence expenditure. This definition of CHE has been widely used in different settings, supporting its value in assessing financial hardship.^11^^,^^12^

### Outcome Variables

The HRQoL was evaluated using the validated Thai version of the EuroQol-5 Dimensions (EQ5D-5L) questionnaire ^29^^,^^30^ The psychometric properties of the EQ5D-5L have been well-established globally. Its dimensions and index values correlate well with functional, pain, emotional, mental health, and clinical measures in different conditions.^31^ The Thai version of EQ5D-5L, used in this study, has been validated regarding reliability and responsiveness and has solid psychometric properties, including internal consistency and construct validity.^30^ The EQ5D-5L comprised 2 parts. In the first part, participants were asked to rate their health state from 1-5 against 5 domains (mobility, self-care, usual activities, pain/discomfort, and anxiety/depression) across 5 levels of severity: 1 no problems, 2 slight problems, 3 moderate problems, 4 severe problems, and 5 extreme problems. The severity scores from all dimensions were combined and calculated to the utility score, which ranged from −1.0 to 1.0. Each EQ5D5L dimension was also recategorized into binary categories (severe vs not severe) based on the disturbance severity, with 1-3 rated as not severe, whereas scores 4 and 5 were classified as severe. The second part of EQ5D-5L comprised the EQ visual analog scale (EQ-VAS), in which participants rate their health on a vertical scale ranging from the best health you can imagine (100) to the worst health you can imagine (0).

### Statistical Analysis

Categorical data were shown as numbers and percentages and compared using the χ^2^ or Fisher exact test. Continuous variables were shown as mean with standard deviations or median with interquartile range and compared using one-way analysis of variance or the Kruskal-Wallis test, as appropriate.

Instead of the linear regression model, which is violated by many assumptions of ordinary least square criteria, and the utility outcomes were in fractional patterns, the fractional regression model was used to determine the effect of CHE on utility. The conditional mean and 95% confidence interval (CI) of utility were presented according to the fractional regression model. We adjusted the effect of CHE on utility by controlling for the following covariates: age (continuous), sex (men/women), types of health schemes (UCS/SSS/CSMBS), groups of CKD (CKD15-60/CKD15/PD/HD), and comorbid conditions (diabetes [yes/no] and cardiovascular disease [yes/no]). In addition, we performed the multivariable linear regression analysis substituting the out-of-pocket/capacity-to-pay ratio for CHE.

We performed a multivariable logistic regression analysis to determine the association between CHE and the severity of disturbing quality of life in each EQ5D5L dimension. The predicted percentage and 95% CI were calculated using the Delta-Margin method and the adjusted logistic model using the same covariates as above. All analyses were conducted using STATA 17.1, and *P* *>* 0.05 was used to determine statistical significance.

## Results

### Patient Characteristics

We excluded 2 patients with incomplete expenditures data and 13 patients who were entirely self-paying from the initial participant pool (n = 1,239). (Fig S1) The study included a total of 1,224 patients (CKD15-60 [n = 435], CKD15 [n = 213], PD [n = 257], and HD [n = 319]). (Table 1) There were 44% under the UCS, 9% under the SSS, and 47% under the CSMBS health schemes. Twenty percent (n = 245) experienced CHE. The proportion with CHE was different between CKD groups (*P* < 0.001), with patients treated with dialysis being higher than nondialysis CKD and HD being higher than PD. The prevalence of diabetes was not different between patients with and without CHE. When compared with patients without CHE, more patients with CHE had cardiovascular disease.

**Table 1:** Demographic and Clinical Characteristics by CHE Status

Characteristics	Total	Not CHE	CHE	*P*
No. of study participants	1,224 (100.0)	979 (80.0)	245 (20.0)	
**Demographic variables**				
Age	63.8 (14.3)	63.5 (14.3)	65.0 (14.1)	0.13
Female	538 (44.0)	424 (43.3)	114 (46.5)	0.36
Health insurance schemes				0.43
UCS	540 (44.1)	434 (44.3)	106 (43.3)	
SSS	109 (8.9)	82 (8.4)	27 (11.0)	
CSMBS	575 (47.0)	463 (47.3)	112 (45.7)	
**Clinical characteristics**				
Nondialysis CKD GFR^a^ (mL/min/1.73m^2^)	23.3 (12.6-37.0)	23.2 (12.4-36.8)	24.9 (13.5-41.0)	0.44
CKD groups				<0.001
CKD 30-60	239 (19.5)	217 (22.2)	22 (9.0)	
CKD 15-29	196 (16.0)	175 (17.9)	21 (8.5)	
CKD <15	213 (17.5)	198 (20.2)	15 (6.1)	
PD	257 (21.0)	200 (20.4)	57 (23.3)	
HD	319 (26.0)	189 (19.3)	130 (53.1)	
Diabetes	552 (45.1)	451 (46.1)	101 (41.2)	0.17
Cardiovascular disease	188 (15.4)	132 (13.5)	56 (22.9)	< 0.001

*Note:* Data as mean±SD, median (25,75 percentile) or n (%).

Abbreviations: CHE, catastrophic health expenditure; CKD, chronic kidney disease; CSMBS, civil servant medical benefit scheme; GFR, glomerular filtration rate; HD, hemodialysis; PD, peritoneal dialysis; SSS, social security scheme; USS, universal coverage scheme.

a GFR includes only individuals not treated with dialysis.

### Utility Scores in Patients With and Without CHE

In the unadjusted analysis, the utility of patients with CHE was significantly lower than those without (*P* < 0.001). (Table 2) After adjusting for the CKD group, age, sex, health insurance schemes, diabetes, and cardiovascular disease, CHE was significantly associated with the utility score. (Table 3)

**Table 2:** Utility Score and Proportion With Severe Impairment for Each EQ5D5L Dimension by CHE Status

Characters	Total	Not CHE	CHE	*P*
No. of patients (%)	1,224 (100.0)	979 (80.0)	245 (20.0)	
Mean utility	0.81 ± 0.21	0.83 ± 0.19	0.75 ± 0.24	<0.001
Median utility	0.88 (0.76-0.94)	0.88 (0.79-0.94)	0.81 (0.72-0.89)	<0.001
**Severe group**				
Mobility, n (%)	143 (11.7)	97 (9.9)	46 (18.8)	<0.001
Self-care, n (%)	46 (3.8)	31 (3.2)	15 (6.1)	0.03
Usual activity, n (%)	95 (7.8)	64 (6.5)	31 (12.7)	< 0.001
Pain/discomfort, n (%)	87 (7.1)	63 (6.4)	24 (9.8)	0.07
Anxiety/depression, n (%)	41 (3.3)	32 (3.3)	9 (3.7)	0.75

*Note:* Data as mean±SD, median (25,75 percentile), or n (%).

Abbreviations: CHE, catastrophic health expenditure.

**Table 3:** Multivariable-Adjusted Association Coefficient Between Either CHE or Out-of-Pocket/Capacity to Pay Ratio With EQ5D-5L Utility Score

Variable	Coefficient	*P*	95% Confidence Interval
CHE^a^	−0.384	< 0.001	−0.577 to −0.190
Out-of-pocket/capacity-to-pay ratio^a^	−0.021	0.002	−0.033 to −0.008

CHE, catastrophic health expenditure; CKD, chronic kidney disease; EQ5D-5L, EuroQol-5 dimensions.

a Adjusted with groups of CKD, age, sex, health insurance schemes, diabetes, and cardiovascular disease.

**Figure 1:**
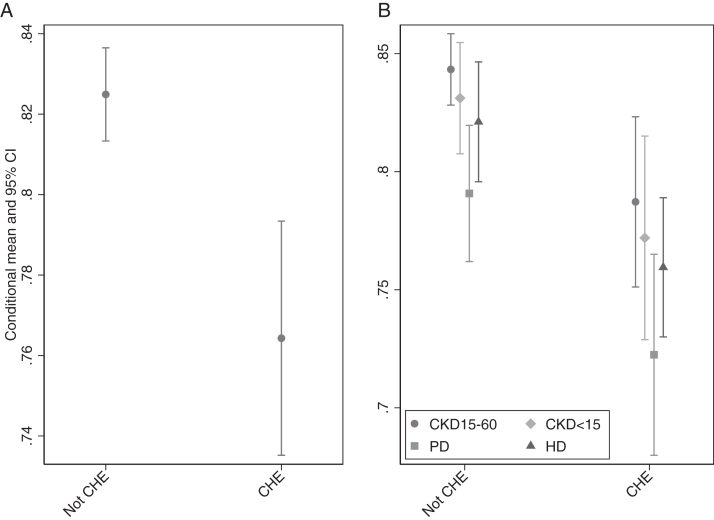
(A) Adjusted utility score according to CHE status. Adjusted for groups of CKD, age, sex, health insurance schemes, diabetes, and cardiovascular disease. (B) Adjusted utility score according to CHE and CKD groups. Adjusted for groups of CKD, age, sex, health insurance schemes, diabetes, and cardiovascular disease. CHE, catastrophic health care expenditures; CKD, chronic kidney disease.

The adjusted conditional mean (95% CI) utility for patients with CHE was lower than those without CHE. (adjusted utility: CHE, 0.76 (0.74-0.79) vs No CHE, 0.82 (0.81-0.84), *P* < 0.001. (Fig 1A)

Figure 1B shows the adjusted utility score according to CKD groups by CHE status. After adjusting for age, sex, health insurance schemes, diabetes, and cardiovascular disease, the adjusted utility score tended to be lower in dialysis than in nondialysis CKD, with PD having the lowest adjusted utility score.

### CHE and Individual EQ5D5L Dimension

Table S1 shows the distribution of EQ5D5L levels for each dimension. In the unadjusted analysis, there were significant differences in severity between CHE and no CHE for mobility, self-care, usual activity, and pain/discomfort, but not anxiety/depression domains. The dimensions of EQ5D5L were dichotomized according to severity into 2 categories (severe vs nonsevere problems). Overall, the prevalence of severe problems was mobility (11.7%), self-care (3.8%), usual activity (7.8%), pain/discomfort (7.1%), and anxiety/depression (3.3%) (Table 2). In the unadjusted analysis, the frequency of severe problems was higher in CHE when compared with Not CHE in terms of mobility, self-care, and usual activity.

**Figure 2:**
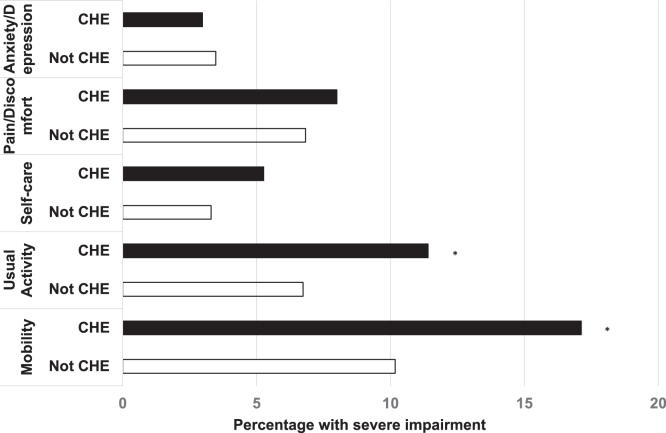
The percentage of patients with severe impairment in each dimension of EQ5D5L according to CHE status. Adjusted with groups of CKD, age, sex, health insurance schemes, diabetes, and cardiovascular disease. CHE, catastrophic health expenditure; CKD, chronic kidney disease. ∗*P* < 0.05,

After multivariable adjustments, CHE had a significantly higher proportion of severe problems in the mobility and usual activity dimensions (Fig 2). The multivariable odds ratio (95% CI) for severe problems in CHE compared with non-CHE were: (mobility: CHE, 1.89 [1.23-2.91], *P* = 0.004; usual activity: CHE, 1.82 [1.10-3.02], *P* = 0.020 (Table 4).

**Table 4:** Multivariable-Adjusted Risk of CHE on Severe Impairment in Each EQ5D5L Dimension

EQ5D5L Dimension	Odds Ratio	*P*	95% Confidence Interval
**Mobility**			
CHE	1.89	0.004	1.23-2.91
**Usual activity**			
CHE	1.82	0.02	1.10-3.02
**Self-care**			
CHE	1.68	0.15	0.83-3.37
**Pain/discomfort**			
CHE	1.19	0.53	0.69-2.06
**Anxiety/depression**			
CHE	0.85	0.70	0.38-1.92

*Note:* Adjusted with groups of CKD, age, sex, health insurance schemes, diabetes, cardiovascular disease.

Abbreviations: CHE, catastrophic health expenditure; CKD, chronic kidney disease.

### Associations of Utility With Out-of-Pocket /Expenditures Ratio

In addition to CHE (defined as out-of-pocket/capacity to pay ratio >40%) as a category, we also explored the relationship of the out-of-pocket/expenditures ratio with utility as a continuum. After multivariable variable adjustments, we found a significant negative association between utility score and out-of-pocket/capacity-to-pay ratio. (Table 3)

### Visual Analog Score

In addition to the utility score, we also evaluated the VAS score, a subjective score documenting how patients feel about their health overall. Similar to the utility score, the VAS score was significantly lower in CHE **(**Table S2)

## Discussion

In this multicenter, nationwide study in Thailand, we examined the relationship between patient-derived financial hardship and health-related quality of life in 1,224 patients with CKD from nondialysis to dialysis. The proportion of CHEs among our patients with CKD was much higher than the general Thai population (2.1%),^32^ or developed countries (0.5%-3 %),^33^ highlighting the burden of CKD in LMICs even in the presence of universal health coverage. The novel findings were that patients with CKD with CHEs experienced worse EQ5D5L utility scores than those without, even after adjusting for covariates. Notably, patients with CKD with CHEs faced a significantly greater risk of severe problems in mobility and activity impairment. At the same time, no significant association was observed for self-care, pain/discomfort, or anxiety/depression domains after multivariable adjustments.

The concept of financial risk protection, originating from economics and insurance theory, has been extensively studied in health system research.^9^ Although out-of-pocket health spending has been shown to affect the nonmedical spending of households, there are fewer studies on the associations of financial burden with clinical outcomes or quality of life.^12^ In patients with cancer, CHEs were found to be associated with increased mortality at 12 months.^24^ In a survey of the Korean general population, Kang et al^25^ found that individuals with CHE exhibited lower EQ-VAS index scores, with patients with noncommunicable diseases being worse off. So far, few studies have explored the relationship of financial burden with clinical outcomes in CKD. Jose et al^26^ demonstrated an association between catastrophic health expenditures and poorer HRQoL, as measured by the EQ5D5L utility score, among 152 patients treated with hemodialysis in India, most of whom were privately funded. However, this study did not explore various dimensions of EQ5D5L and excluded patients with nondialysis CKD or peritoneal dialysis.^26^ Our multicenter study expanded on existing data by investigating the relationship between CHEs and HRQoL across the CKD spectrum, from nondialysis to HD and PD, in Thailand. After adjusting for multiple covariates, our findings showed that patients with CHEs had lower utility scores. These results were consistent with the patient’s perspective, as reflected in the EQ5D5L VAS score.

Our findings are consistent with previous data examining socioeconomic indices on patient quality of life. Lower education and income were negatively associated with reduced quality of life in nondialysis patients with CKD from India and Brazil.^23^^,^^34^ Recent meta-analyses found that lower socioeconomic status, measured by income and occupation, was associated with higher mortality or overall symptom burden in patients receiving maintenance dialysis.^35^^,^^36^

Because this study is cross-sectional, the causal relationship between CHEs and quality of life cannot be determined. The association between financial catastrophe and severe mobility and activity impairment suggests a possible 2-way interaction wherein economic strain and poor health may exacerbate each other in a vicious cycle.^37^ For instance, high out-of-pocket expenses for transportation and caregivers resulting from mobility impairment may contribute to CHE. Alternatively, financial burdens may limit patients’ ability to afford a healthy diet, medications, or dialysis access, potentially worsening their conditions.

The lack of association between catastrophic health expenditures and severe pain/discomfort or anxiety/depression observed in our study differed from previous studies in nondialysis CKD from India or from a meta-analysis of several HD populations, where socioeconomic indices such as lower education and lower income were negatively associated with depression, fatigue, and pain.^23^^,^^36^ The prevalence of anxiety/depression in our patients was low. Thailand’s universal health care, by covering basic needs such as pain management, mental health support, and emergencies, may reduce psychological stress related to the financial burden of CKD. In contrast, LMICs without adequate public support often face uncertainty in affording health care, leading to increased mental health concerns and untreated symptoms in low-income patients.

This study has several strengths and implications. To our knowledge, this is the first study to assess the association between financial burden and quality of life from the patient’s perspective in a spectrum of CKD. By including patients treated with nondialysis to dialysis in a multicenter countrywide design, this study highlights the significance of using CHEs as an objective indicator of the health-related financial burden of CKD in the context of universal health care coverage in a middle-income country like Thailand. Although previous health system research studies have explored associations of socioeconomic statuses, such as income, with HRQoL, this study uniquely focuses on the specific financial burden imposed by CHEs derived directly from the patient’s perspective. The observed negative impact of the financial catastrophe on HRQoL, particularly in mobility and activity domains, highlights the critical relationship between financial protection and the patient’s functional abilities and overall well-being.

This study emphasizes the need for health financing policies addressing the economic vulnerability of patients with CKD in middle-income countries, where financial hardship remains a considerable barrier to optimal care. We previously reported that financial toxicity disproportionately affects the lower income groups, with patients treated with hemodialysis under the UCS facing the greatest burden.^19^ Travel expenses for hemodialysis sessions were the main out-of-pocket cost in HD, whereas other CKD groups also had travel costs for hospital clinic visits, albeit to a lesser degree. Uncovered medical payments also represented major expenses in all CKD groups, with costs increasing as the disease progressed. Integrating targeted financial support into CKD management strategies, especially for the most vulnerable patients, is needed to alleviate these financial challenges. Potential interventions include providing financial planning services, transportation subsidies, extending insurance coverage to reduce out-of-pocket medical expenses, and expanding home-based treatment options such as PD.

However, several limitations should be acknowledged. First, the study’s cross-sectional design prevents causal inferences. Second, we recruited consecutive patients from randomly selected periods at each hospital. Although this does not meet strict patient-level randomization, we believe that this method still allows a broad representation of CKD in our population. Nonetheless, recruiting hospital outpatients may have excluded severely ill, bedridden individuals, underestimating CKD’s impact on financial burden and HRQoL. Third, EQ-5D-5L, although effective for broad psychometric comparisons across diseases, may be less sensitive to detect subtle CKD-specific quality-of-life changes, potentially underestimating the impact of financial hardship. Fourth, recall bias might reduce the accuracy of out-of-pocket expenses and CHE reporting despite our efforts to limit the recall period to 6 months and the use of trained personnel to administer a structured questionnaire. Although we used the WHO-recommended 40% threshold of household capacity to pay, this fixed threshold may not fully capture the financial burden in those with fluctuating incomes or health care needs. To minimize threshold bias, we also analyzed the out-of-pocket/capacity-to-pay ratio as a continuous parameter to support our findings. Finally, CHEs do not capture indirect costs such as lost wages or caregiving or reflect accumulated long-term financial burdens. Therefore, although CHE provides a snapshot of financial hardship, it may underestimate broader socioeconomic impacts over time. Future studies should integrate CHE with patient-reported financial distress scales and CKD-specific quality-of-life assessments to fully understand the cumulative effects of financial strain as CKD progresses.

In conclusion, despite universal health coverage, our study highlights the persistence of financial hardship among Thai patients with CKD, particularly those with severe mobility and activity limitations. Future longitudinal studies are necessary to understand better how financial strain impacts mental and physical health across the CKD spectrum as the disease progresses. Such approaches will guide the development of more targeted interventions and policies to reduce financial hardship and improve the quality of life in patients with CKD, especially in middle-income countries.
