# Supplementary material for: Quality of Life in Patients with CKD With Catastrophic Health Care Expenditures: A National Study From Thailand
Source: Kidney Med. 2025 Feb 27;7(5):100987. doi: 10.1016/j.xkme.2025.100987 (PMC12008141; doi:10.1016/j.xkme.2025.100987)
Supplement: Supplementary File (PDF) — Figure S1; Tables S1-S2. [file mmc1.pdf]

Figure S1: Flow of study

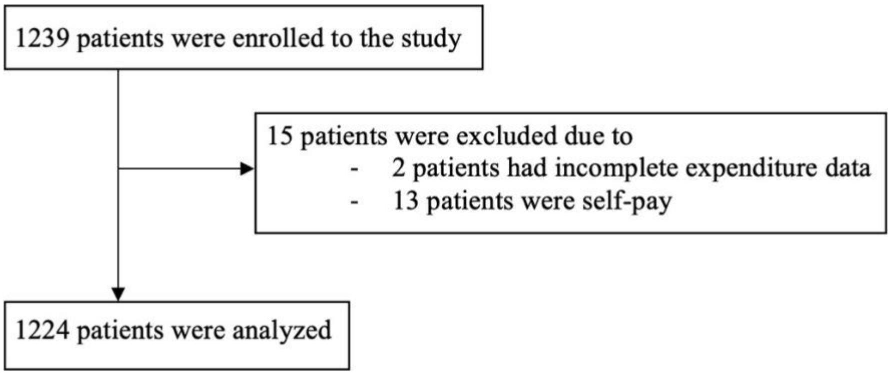

Table S1: Number of patients in each severity level in dimensions of EQ5D-5L by CHE status

Characters	Not CHE	CHE	Total	P-value
n (%)	979 (80.0)	245 (20.0)	1224 (100.0)	
Mobility [a]				**< 0.001**
1	414 (42.3)	54 (22.0)	468 (38.2)	
2	268 (27.4)	75 (30.6)	343 (28.0)	
3	200 (20.4)	70 (28.6)	270 (22.1)	
4	83 (8.5)	36 (14.7)	119 (9.7)	
5	14 (1.4)	10 (4.1)	24 (2.0)	
Self-Care [a]				**0.01**
1	888 (90.7)	206 (84.1)	1094 (89.4)	
2	33 (3.4)	11 (4.5)	44 (3.6)	
3	27 (2.8)	13 (5.3)	40 (3.3)	
4	15 (1.5)	3 (1.2)	18 (1.5)	
5	16 (1.6)	12 (4.9)	28 (2.3)	
Usual Activity [a]				**< 0.001**
1	751 (76.7)	151 (61.6)	902 (73.7)	
2	102 (10.4)	38 (15.5)	140 (11.4)	
3	62 (6.3)	25 (10.2)	87 (7.1)	
4	22 (2.2)	11 (4.5)	33 (2.7)	
5	42 (4.3)	20 (8.2)	62 (5.1)	
Pain/Discomfort [a]				**< 0.001**
1	349 (35.6)	56 (22.9)	405 (33.1)	
2	377 (38.5)	101 (41.2)	478 (39.1)	
3	190 (19.4)	64 (26.1)	254 (20.8)	
4	59 (6.0)	21 (8.6)	80 (6.5)	
5	4 (0.4)	3 (1.2)	7 (0.6)	
Anxiety/Depression [a]				**0.31**
1	618 (63.1)	138 (56.3)	756 (61.8)	
2	247 (25.2)	70 (28.6)	317 (25.9)	
3	82 (8.4)	28 (11.4)	110 (9.0)	
4	31 (3.2)	9 (3.7)	40 (3.3)	
5	1 (0.1)	0 (0.0)	1 (0.1)	

[a] N (%), CHE, Catastrophic health expenditure

Table S2: EQ5D VAS score by CHE status

Characters	Total	Not CHE	CHE	P-value
Mean VAS score	74.15 (14.80)	74.86 (14.71)	71.31 (14.84)	< 0.001
Median VAS score	75.00<br>(70.00. 80.00)	80.00<br>(70.00, 80.00)	70.00<br>(60.00, 80.00)	< 0.001

Data as Mean ±SD, Median (25,75 percentile), CHE, Catastrophic health expenditure
